# Dynamic Magnetic Resonance Imaging (MRI) in Inguinal-Related Chronic Groin Pain (CGP): Comparison With Systematic Surgical Assessment

**DOI:** 10.7759/cureus.55947

**Published:** 2024-03-11

**Authors:** Benjamin Dallaudiere, Hugo Sans, Gilles Reboul, Laurence Dallet, Patricia Reau, Sylvain Bise, Nicolas Bouguennec, Lionel Pesquer

**Affiliations:** 1 Radiology, Clinique du Sport de Bordeaux Mérignac, Bordeaux, FRA; 2 Parietal Surgery, Clinique du Sport de Bordeaux Mérignac, Bordeaux, FRA; 3 Unité mixte de recherche (UMR) 5536, Centre national de la recherche scientifique (CNRS), Bordeaux, FRA; 4 Anatomopathology, Clinique du Sport de Bordeaux Mérignac, Bordeaux, FRA; 5 Orthopedic Surgery, Clinique du Sport de Bordeaux Mérignac, Bordeaux, FRA

**Keywords:** valsalva, dynamic, mri, abdominal wall, groin pain

## Abstract

Objective

This study aimed to assess the performance of dynamic MRI in Chronic Groin Pain (CGP) related to the inguinal region, comparing it with surgery as the gold standard.

Materials and methods

A cohort of 25 consecutive patients exhibiting persistent clinical inguinal-related CGP underwent a pre-surgical pelvis MRI. Imaging encompassed strictly axial Fast Spin Echo (FSE) T1 sequences, both without (static sequence) and with Valsalva Maneuver (VM, dynamic sequence), alongside axial-oblique Proton Density weighted with Fat Saturation (PDFS). Evaluation of these sequences focused on identifying Abdominal Wall (AW) injuries. A consistent surgical approach was employed by the same surgeon across all patients (34 AW injuries in 25 patients). Specificity (Sp), Sensitivity (Se), Negative Predictive Value (NPV), Positive Predictive Value (PPV), and overall accuracy of MRI sequences and their combinations for detecting AW injuries were computed by comparing them to surgical findings.

Results

Ninety sequences were obtained, revealing that the axial PDFS oblique sequence emerged as the most singularly reliable (Accuracy: 58.82%). The optimal sequence combination was found to be axial T1 combined with axial T1 VM, exhibiting an accuracy of 75.00% (Se: 85.71%, Sp: 70.59%, PPV: 54.55%, NPV: 92.31%, with an average duration of 4 minutes and 31 seconds).

Conclusion

Based on our findings, we advocate for the adoption of the axial FSE T1 combined with Valsalva Maneuver as a dependable protocol for inguinal-related CGP, characterized by a highly reasonable examination duration.

## Introduction

Chronic groin pain (CGP, at least 6 weeks) represents a prevalent issue among athletes, notably soccer and rugby players [1,2,3]. Characterized by inguinal-related discomfort during hip twisting and extension [4,5], CGP poses important diagnostic challenges owing to its three clinical entities: Adductor Longus (AL), Pubic Symphysis (PS), and Abdominal Wall (AW) lesions, often intricately intertwined.

Recent investigations and consensus conferences have underscored the crucial role of imaging in CGP management [1]. Ultrasound (US) is widely used for AW assessment due to its real-time dynamic examination capability, while Magnetic Resonance Imaging (MRI) is preferred for PS and AL assessment [1,2]. However, existing literature suffers from limitations, including small sample sizes, inconsistency in imaging protocols, variations in MR scanner types, and a lack of uniformity in surgical approaches [3,4,5].

Recent studies have demonstrated a noteworthy correlation between MRI findings and surgical outcomes, particularly in cases involving AL tendinopathy and deep inguinal canal dehiscence [4,6]. Ducouret et al. recently assessed the value of MRI sequences in CGP diagnosis, focusing on AW and AL injuries and systematically comparing them to surgery as the gold standard. They propose a simplified pelvis MRI protocol comprising a 4-sequence cluster for CGP: axial T1 - axial Proton Density weighted with Fat Saturation (PDFS) - sagittal PDFS - coronal T1, offering a reasonable examination time [7].

Regrettably, no prior study has specifically examined the reliability of static and dynamic MRI sequences compared to AW surgical and anatomopathological assessment. Hence, our objective was to evaluate the diagnostic reliability of dynamic MRI sequences in inguinal-related CGP, considering AW injury, and systematically comparing the results with surgery as the gold standard

## Materials and methods

Patient cohort

A retrospective study was conducted at a single center, involving 25 consecutive athletes (21 males and four females) from January 2016 to October 2020. All participants were referred to our radiology department by a specialized surgeon (GR) in CGP surgery for a pre-abdominal wall (AW) surgical MRI. Among them, five patients presented with AW inguinal-related right-side pain, 11 with AW inguinal-related left-side pain, and complained of AW inguinal-related bilateral pain, resulting in a total of 34 AW injuries in 25 patients. The mean age of the study participants was 40.12 years (from 27.12 to 53.12 years old). ​​​ Informed consent was obtained from all participants. The study received approval from the local ethic board (“Comité de Protection des Personnes”: CPP SOOM III, registration number DC 2015/109).

Inclusion and Exclusion Items

Inclusion criteria encompassed clinical CGP resistant to medical interventions, with pain defined as lasting more than 6 weeks and assessed by a General Practitioner (GP) with ineffective medical treatment and eccentric exercises in physiotherapy. The mean pain time before MRI was 8.2 months (± 0.7 months). Exclusion criteria comprised contraindications to MRI, age below 18 years, and prior pubic surgery.

MRI protocol

All participants underwent pelvis MRI using the same 1.5-Tesla MR scanner All participants underwent pelvis MRI using the same 1.5-Tesla MR scanner (General Electrics (GE) ® Healthcare) with a cardiac coil (16 channels, 32 elements). The following sequences were performed: axial-oblique (in symphysis plane) Proton Density weighted with Fat Saturation (PDFS), axial Fast Spin-Echo T1-weighted (FSE T1), axial FSE T1 with Valsalva Maneuver (VM): (a) Axial fast spin-echo T1-weighted (FSE T1) (TR (repetition time)/TE (echo time): 568/ min full ms, Field of View (FOV): 36 cm, matrix: 640 × 384, 16 slices). Slice thickness: 4.5 mm; number of excitations: two; intersection gap: 1 mm; length: 1.55 min. (b) Coronal FSE T1 (TR/TE: 724/ min full ms; FOV: 38 cm; matrix: 512 × 320, 20 slices). Slice thickness: 3.5 mm; number of excitations: three; intersection gap: 1 mm; length: 2.29 min. (c) Axial-oblique (in symphysis plane) proton density weighted with fat saturation (PDFS) (TR/TE: 2465/58 ms; FOV: 26 cm; matrix: 416 × 288; 15 slices). Slice thickness: 3.5 mm; number of excitations: six; intersection gap: 0.7 mm; length: 2.36 min. (d) Coronal PDFS (TR/TE: 3511/38 ms; FOV: 38 cm; matrix: 512 × 320; 20 slices). Slice thickness: 4 mm; number of excitations: four; intersection gap: 1 mm; length: 2.42 min. (e) Sagittal PDFS (TR/TE: 1954/49 ms, FOV: 48 cm, matrix: 384 × 288, 13 slices). Slice thickness: 4 mm; number of excitations: five; intersection gap: 1 mm; length: 3.36 min. (f) Axial FSE T1 with fat saturation and gadolinium enhancement (FSGE) (TR/TE: 506/min full ms; FOV: 36 cm; matrix: 512 × 320; 16 slices). Slice thickness: 4.5 mm; number of excitations: 2; intersection gap: 1 mm; length: 1.42 min. (g) Coronal FSE T1 FSGE (TR/TE: 732/min full ms, FOV: 38 cm, matrix: 512 × 320; 20 slices). Slice thickness: 4 mm; number of excitations: two; intersection gap: 1 mm; length: 2.12 min. (h) Axial FSE T1 with Valsalva maneuver (VM) in dubious cases to sensitize parietal insufficiency detectability (TR/TE: 568/min full ms; FOV: 36 cm; matrix: 640 × 384, 16 slices). Slice thickness: 4.5 mm; number of excitations: two; intersection gap: 1 mm; length: 1.55 min.

MRI analysis

Each MRI sequence was evaluated by a musculoskeletal (MSK) radiologist (HS: 1-year MSK experience, after CGP MRI training on not included patients with a senior MSK radiologist, and BD: 10 years post-residency experience), who was blinded to patient information, except for the pain side. A minimum delay of 3 weeks was observed between each sequence interpretation.

All images were analyzed using GE® Advantage Workstation 4.5 (GE Healthcare, Chicago, USA).

The MRI images were scrutinized for AW assessment, considering: the presence or absence of inguinal or femoral hernia, fat involvement and trophicity in Rectus Abdominis (RA), presence or absence of a Transversalis Fascia (TF) bulging, defined by an anterior convexity, inguinal orifice diameter (measured at level of the emergence of the inferior epigastric vessels).

An AW was deemed injured if the inguinal orifice diameter exceeded 20 mm or if TF bulging was observed on static and/or dynamic sequences. AW disease severity was graded as follows: Grade 0: Normal abdominal wall, Grade 1: Inguinal orifice < 2 cm, Grade 2: Inguinal orifice between 2 and 3 cm, Grade 3: Inguinal orifice between 3 and 4 cm, Grade 4: Inguinal orifice > 4 cm.

**Figure 1:**
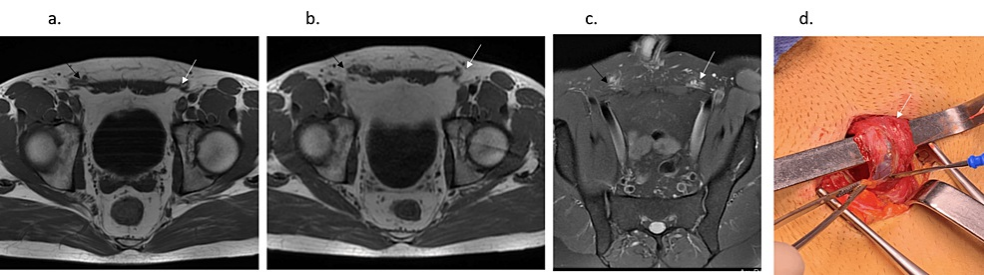
A 33-year-old man, professional soccer player (Second league) with bilateral pain. Axial T1 in Abdominal Wall (AW)-related Chronic Groin Pain (CGP) (a) and Axial oblique Proton Density weighted with Fat Saturation (PDFS) in AW (c), with bilateral bulging (bilateral fatty overload): grade 3 in right inguinal orifice (black arrow) and grade 2 in left inguinal orifice (white arrow) in these static MRI sequences. This AW bilateral lesion becomes grade 4 in the right inguinal orifice and grade 3 in the left inguinal orifice in axial T1 VM (b) (same patient, same level). Left AW exposed in AW-related CGP with black arrow = spermatic cord (covered by external oblique aponeurosis).

**Figure 2:**
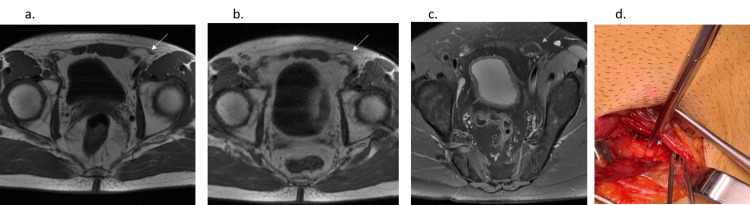
A 28-year-old man, professional tennis player with left pain. Axial T1 in AW-related Chronic Groin Pain (CGP) (a) and Axial oblique Proton Density weighted with Fat Saturation (PDFS) in Abdominal Wall (AW) (c) with bulging (fatty overload): grade 3 in left inguinal orifice (white arrow) in these static MRI sequences. This AW lesion becomes grade 4 in the left inguinal orifice in axial T1 VM (b) (same patient, same level). Left AW exposed in AW-related CGP (before the Shouldice technique) with black arrow = weakness at conjoint tendon insertion on left Raectus Abdominis.

Figure 1 and Figure 2 show examples of AW MRI and surgery assessment.

Surgical intervention

**Figure 3:**
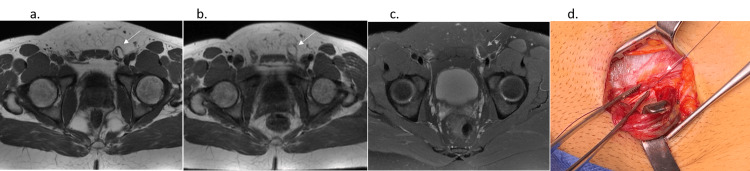
A 25-year-old man, soccer player (First League) with left pain. Axial T1 in Abdominal Wall (AW)-related Chronic Groin Pain (CGP) (a) and Axial oblique Proton Density weighted with Fat Saturation (PDFS) in AW (c) with left inguinal hernia in left inguinal orifice (white arrow) in these static MRI sequences. The important TF bulging increase in Valsalva Maneuver (VM) in axial T1 VM (b) (same patient, same level). Left AW exposed in AW-related CGP with white arrow = Prolene sutures (lowering to the inguinal ligament). The surgical clamp shows the inferior margin of an external oblique aponeurosis.

All surgical procedures were conducted by the same specialized surgeon (GR) with expertise in CGP. The mainstream suture-based repair employed was the Shouldice technique, characterized by a four-layer reconstruction of the TF using Prolene sutures (Ethicon, Cincinnati, USA), coupled with a conjoint tendon lowering to the iliopubic tract (1,3). Throughout the surgical intervention, the surgeon consistently evaluated and graded the Abdominal Wall (AW). Similar to the criteria used in MRI assessment, the AW was considered injured if the surgeon observed an inguinal orifice diameter exceeding 2 cm or if a Transversalis Fascia (TF) bulging was identified (Figure 3).

Histological analysis was performed for one case by the surgeon. The tissue specimen was fixed in a 10% formalin solution for 24 hours and subsequently embedded in paraffin. Three-micron-thick sections were cut and stained with hematoxylin and eosin.

Data and statistical analysis

All statistical analyses were performed with R software (R Core Team, R Foundation for Statistical Computing, Vienna, Austria). We evaluated the performance of the MRI sequence alone and in combination with AW assessment with a t-test. Non-operated AWs were asymptomatic and then considered clinically normal. The significance threshold selected for all the statistical analyses was 0.05. The results from the quantitative variables are presented as mean ± Standard Deviation (SD), minimum, maximum, and median values. The analysis was made on the basis of 34 AWs assessed, considering right and left sides in 25 patients. Specificity, Sensitivity, Positive Predictive Value, Negative Predictive Value, and Accuracy were calculated. Accuracy is defined as the ability to differentiate affected or unaffected patients correctly (Formula: (True Positives + True Negatives)/All). All calculations were made using data from surgery (AW assessment) as the gold standard and the diagnosis was made on the basements of MRI sequences which were studied as the other variable.

## Results

Surgical data

The surgeon performed 34 abdominal wall (AW) surgeries on 25 patients: five on the right side, 11 on the left side, and nine bilaterally. 

Throughout the surgical procedures, 14 right AW lesions and 20 left AW lesions were identified, comprising 10 grade 1 lesions, 14 grade 2 lesions, 10 grade 3 lesions, 18 with transverse fascia (TF) bulging, and two with inguinal hernia.

Magnetic Resonance Imaging (MRI) analysis with surgical comparison

A total of 90 sequences were acquired from 25 patients, including 32 axial T1, 24 axial T1 volumetric magnetization (VM), and 34 axial oblique proton density fat saturation (PDFS) sequences. The MRI findings are summarized in Table 1.

**Table 1:** MRI findings in AW assessment N = number of sequences; Ax = Axial; RMIOD = Right Mean Inguinal Orifice Diameter (in mm (mean (SD)); LMIOD = Left Mean Inguinal Orifice Diameter (in mm (mean (SD)); RTFB = Right Transversalis Fascia Bulging; LTFB = Left Transversalis Fascia bulging; RRAA = Right Rectus Abdominis Atrophy; LRAA = Left Rectus Abdominis Atrophy; RAWI = Right Abdominal Wall Injury; LAWI = Left Abdominal Wall Injury; PDFS = Proton Density weighted with Fat Saturation; SD = standard deviation; AW = abdominal wall; T1 VM = T1 volumetric magnetization (VM).

		RTFB	LTFB	RRAA	LRAA	RAWI	LAWI	RMIOD	LMIOD
SEQUENCES	N								
Ax T1	32	2	4	13	19	6	6	19.1 (6.8)	18.8 (6.8)
Ax T1 VM	24	7	11	9	15	8	11	24.2 (7.9)	23.0 (7.8)
Ax oblique PDFS	34	1	2	14	20	10	8	20.9 (5.9)	20.4 (6.2)
TOTAL	90	10	17	36	54	24	25	20.7 (7.3)	19.2 (8.5)

Primary findings for AW assessment

In a binary classification (normal vs. injured), 12 out of 32 (37.50%) AW were considered abnormal in axial T1, 19 out of 24 (79.17%) in axial T1 VM, and 18 out of 34 (52.94%) in axial oblique PDFS (total average: 49 out of 90; 54.44%).

Sequence With Only Static T1 Assessment

The accuracy of the axial T1 sequence (n=32) reached 56.25% (95% CI [37.6-73.6]), correctly predicting 18 AW out of 32 in 24 patients. Four out of 10 AW were true positives (sensitivity (Se): 40.00%, 95% CI 23.1-56.9), and 14 out of 22 AW were true negatives (specificity (Sp): 63.64%, 95% CI 46.9-80.3). The positive predictive value (PPV) and negative predictive value (NPV) were 33.33% (95% CI 5.1-61.6) and 70.00% (95% CI 54.1-85.8), respectively. Among the findings, five AW had TF bulging, all AW exhibited RA trophic, 31 AW had RA IG, and 2 AW with hernia were detected. The mean inguinal orifice diameter was 18.8 mm (SD: 6.7).

Sequence With Only Axial Oblique PDFS Assessment

With 34 AW assessed, the axial PDFS had the highest accuracy at 58.82% (95% CI 40.7-75.3), correctly predicting 20 AW. Seven out of 10 AW were true positives (Se: 70.00%, 95% CI 54.6-85.4), and 13 out of 24 AW were true negatives (Sp: 54.17%, 95% CI 37.4-70.9). PPV and NPV were 38.89% (95% CI 22.5-55.3) and 81.25% (95% CI 74.8-87.8), respectively. Three AW with TF bulging were found, all AW exhibited RA trophic and RA IG, and 2 AW with hernia were identified. The mean inguinal orifice diameter was 20.4 mm (SD: 6.1).

Sequence With Only Dynamic T1 Assessment

The accuracy of the axial T1 VM sequence (n=24) was 41.67% (95% CI 22.11-63.36), correctly predicting 10 AW out of 24 in 17 patients. Six out of seven AW were true positives (Se: 85.71%, 95% CI 71.7-99.7), and four out of 17 AW were true negatives (Sp: 23.53%, 95% CI 6.5-40.4). Additionally, PPV and NPV were 31.58% (95% CI 12.9-50.2) and 80.00% (95% CI 63.9-96). Among the findings, 18 AW had TF bulging, all AW exhibited RA trophic and RA IG, and two AW with hernia were detected. The mean inguinal orifice diameter was 23.0 mm (SD: 7.8).

Sequences Combination With T1 Static and Dynamic Assessment

The accuracy of the axial T1 static with dynamic sequence was 75% (95% CI 53.3-90.2), correctly predicting 18 AW out of 24 in 17 patients. Six out of 10 AW were true positives (Se: 85.71%, 95% CI 71.7-99.7), and 12 out of 17 AW were true negatives (Sp: 70.59%, 95% CI 39.1-78.5). PPV and NPV were 54.55% (95% CI 34.6-74.5) and 92.31% (95% CI 81.7-102.9), respectively.

Sequences Combination With T1 Static and PDFS Assessment

The accuracy of axial PDFS with axial T1 fat-saturated gadolinium-enhanced (FSGE) (n=32) was 65.62% (95% CI 46.8-81.4), correctly predicting 21 AW out of 32 in 24 patients. Seven out of 10 AW were true positives (Se: 70.00%, 95% CI 51.6-88.3), and 14 out of 22 AW were true negatives (Sp: 63.64%, 95% CI 44.4-82.9). PPV and NPV were 46.67% (95% CI 26.7-66.6) and 82.35% (95% CI 67.1-97.6), respectively.

Sequences Combination With T1 Dynamic and Static PDFS Assessment

The accuracy of axial PDFS with axial T1 dynamic (n=24) was 70.83% (95% CI 48.91-87.38), correctly predicting 17 AW out of 24 in 17 patients. Six out of seven AW were true positives (Se: 85.71%, 95% CI 71.7-99.7), and 11 out of 17 AW were true negatives (Sp: 64.71%, 95% CI 45.6-83.8). PPV and NPV were 50.00% (95% CI 30.00-70.0) and 91.67% (95% CI 80.6-102.7), respectively.

Sequences Combination With T1 Static/Dynamic and Static PDFS Assessment

The accuracy of axial PDFS with axial T1 static/dynamic (n=24) was the highest at 75% (95% CI 40.6-81.2), correctly predicting 18 AW out of 24 in 17 patients. Six out of seven AW were true positives (Se: 85.71%, 95% CI 77.6-93.8), and 12 out of 17 AW were true negatives (Sp: 70.59%, 95% CI 52.4-88.8). PPV and NPV were 54.55% (95% CI 34.6-74.4) and 92.31% (95% CI 81.7-102.9). The main MRI diagnostic accuracy results are presented in Table 2.

**Table 2:** Summarizing the comparison of MRI sequences with surgery in AW assessment. N = number of sequences; Ax = Axial; Cor = Coronal; Sag = Sagittal; Se = Sensitivity; Sp = Specificity; PPV = Predictive Predictive Value; NPV = Negative Predictive Value; Acc = Accuracy, PDFS = Proton Density weighted with Fat Saturation, T1 VM = T1 volumetric magnetization. Se (sensitivity), Sp (specificity), PPV (positive predictive value), NPV (negative predictive value), and Acc (accuracy) data are in %.

	Sequences	N	Se	Sp	PPV	NPV	Acc
1 SEQUENCE	Axial T1	32	40.00	63.64	33.33	70.00	56.25
1 SEQUENCE	Axial T1 VM	24	85.71	23.53	31.58	80.00	41.67
1 SEQUENCE	Axial PDFS oblique	34	70.00	54.71	38.89	81.25	58.82
2 SEQUENCES	Axial T1 + Axial T1 VM	24	85.71	70.59	54.55	92.31	75.00
2 SEQUENCES	Axial T1 + Axial PDFS oblique	32	70.00	63.64	46.67	82.35	65.62
2 SEQUENCES	Axial T1 VM + Axial PDFS oblique	24	85.71	64.71	50.00	91.67	70.83
3 SEQUENCES	Axial T1 + Axial T1 VM + Axial PDFS oblique	24	85.71	70.59	54.55	92.31	75.00

Histological evaluation

**Figure 4:**
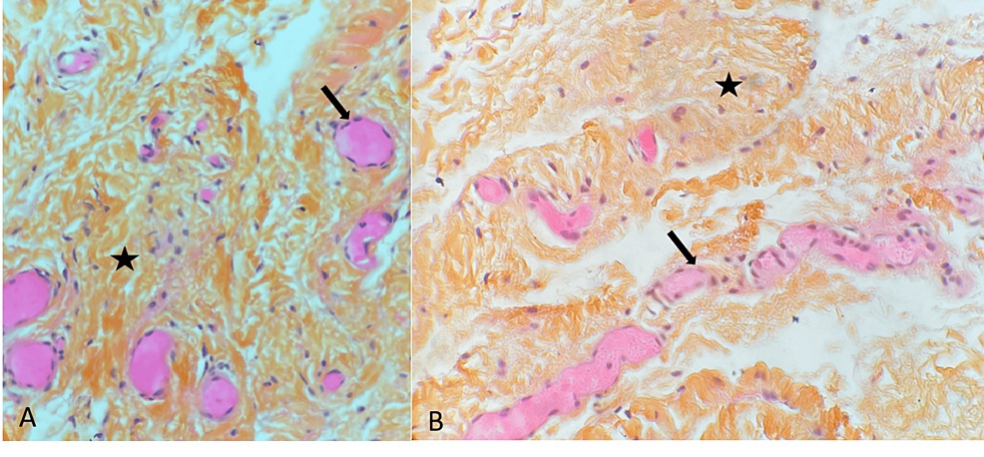
A 26-year-old man, professional Rugby player (First league). Histology in pathologic fascia transversalis (a: magnification x10, b: magnification x40) with vascular congestion - neovascularization (black arrow) with collagen fiber disorganization (black star).

The histological assessment of one case revealed signs of vascular congestion and neovascularization, accompanied by disorganization of collagen fibers. Notably, there were no observed macrophages or multinucleated cells. Additionally, the examination did not identify hematic deposits, calcification, or a fibroblast repair reaction (Figure 4).

## Discussion

The most effective sequence combination identified was axial T1 combined with axial T1 VM, exhibiting an accuracy of 75.00%. Based on these findings, we propose the adoption of axial Fast Spin Echo (FSE) T1 combined with Valsalva Maneuver as a reliable protocol for the assessment of CGP, offering a very reasonable imaging duration.

Axial Proton PDFS in the symphysis plane emerged as the most reliable single sequence for AW assessment. The use of this oblique plane, with a smaller Field of View (FOV), facilitated the discrimination of AW lesions in alignment with the findings of Omar et al [4].

Axial T1 VM demonstrated promise as an efficient sequence for assessing inguinal orifices bilaterally. The utilization of a larger FOV enabled the comparison of bilateral parietal components, while dynamic abdominal pressure sensitized Abdominal Wall Transversalis Fascia (AW TF) bulging, akin to ultrasound (US) practices [8-15]. Ultimately, this shortened 4-minute 31-second protocol allowed for a precise assessment of AW by minimizing patient movements, which are a significant source of artifacts that can impede interpretation.

In the Omar et al article., the MRI specificity and sensitivity for CGP injury detection were reported with excellent results for evaluation concerning both wall and musculo-tendinous structures. However, Omar et al.'s study had limitations, including MR imaging from different centers with varied techniques and protocols. Their control group was smaller than their patient group and didn't match for athletic activity, age and sex patients. Additionally, this series did not describe AW patterns in surgical and MRI protocols [4].

In the Larbi et al. series, specificity, sensitivity, Negative Predictive Value (NPV), and Positive Predictive Value (PPV) of MRI for AW injuries were not reported. While this pilot study demonstrated a good correlation between MRI and surgery for the inguinal canal, there was a low correlation between MRI and surgery when attempting to grade these lesions. Nevertheless, there was an important correlation when considering only the item "affected versus unaffected" (73% for AW and 100% for AL tendon). Despite systematic histology comparison, Larbi et al.'s study faced limitations, primarily stemming from the small size matched for sports activity, age, and sex [6].

In the Ducouret et al. study [7], the overall accuracy in the Abdominal Wall (AW) assessment (63.42%) was lower than the findings reported by Larbi et al. (73%) [6]. It's essential to note that this discrepancy takes into account a different analysis method and a larger population, which could potentially be more reflective of real Magnetic Resonance Imaging (MRI) reliability. This study specifically examined the performance of MRI sequences, both independently and in combination, for Chronic Groin Pain (CGP) diagnosis, with a systematic comparison to surgery. The accuracy reached 80.20% for Adductor Longus (AL) and 63.42% for AW. Coronal T1 Fast Spin Echo (FSGE) and axial T1 with Valsalva Maneuver (VM) were identified as the most individually reliable sequences (Accuracy: 91.67% in AL and 83.33% in AW). Comparisons with published literature led the authors to propose an MRI protocol for AL and AW assessment using a four-sequence cluster: coronal T1 combined with axial Proton Density weighted with Fat Saturation (PDFS) (in Pubic Symphysis plane), sagittal PDFS, and axial T1 VM. This four-sequence protocol, excluding Gradient Echo (GE), demonstrated broad acceptability in daily practice, with an accuracy of 77.78%, Sensitivity of 100%, Specificity of 69.23%, Positive Predictive Value (PPV) of 55.56%, and Negative Predictive Value (NPV) of 100%, all within a very reasonable average duration of 10 minutes and 36 seconds [7].

Nevertheless, our study has several limitations. Firstly, the patient cohort was relatively small (n = 25). However, to our knowledge, it constitutes the largest homogeneous series focusing on AW assessment with a dynamic MRI, evaluating diagnostic value, and systematically comparing results to surgery. Secondly, the retrospective study design reduces the level of evidence. Nonetheless, all patient assessments were consistently performed on the same MR scanner by the same radiologist and surgeon. Lastly, the abdominal wall, being a collagen-rich structure, lacked a validated histological score for grading AW injury.

## Conclusions

The study delved into the diagnostic value of static and dynamic MRI sequences, either standalone or combined, in CGP diagnosis, specifically considering AW injuries with compared outcomes to surgical assessments. Based on these findings, we recommend adopting axial Fast Spin Echo (FSE) T1 with Valsalva Maneuver as a reliable sequence for inguinal-related CGP, characterized by a very reasonable examination duration.
